# Dose-response relationships of resistance training in Type 2 diabetes mellitus: a meta-analysis of randomized controlled trials

**DOI:** 10.3389/fendo.2023.1224161

**Published:** 2023-09-25

**Authors:** Wanying Su, Meiyi Tao, Lin Ma, Ke Tang, Fang Xiong, Xuan Dai, Yuelan Qin

**Affiliations:** ^1^ Joint Surgery and Sport Medicine Department, Hunan Provincial People's Hospital (The First Affiliated Hospital of Hunan Normal University), Changsha, Hunan, China; ^2^ Nursing Department, Hunan Provincial People's Hospital (The First Affiliated Hospital of Hunan Normal University), Changsha, Hunan, China; ^3^ Endocrinology Department, Hunan Provincial People's Hospital (The First Affiliated Hospital of Hunan Normal University), Changsha, Hunan, China

**Keywords:** resistance training, diabetes, glycated hemoglobin, blood glucose, meta-analysis

## Abstract

**Background:**

Globally, type 2 diabetes mellitus (T2DM) accounts for approximately 90% of diabetes cases. Resistance training (RT) is frequently employed to diminish Glycated Hemoglobin (HbA1c) and Fast Blood Glucose (FBG) levels in T2DM patients. Yet, the specific dose-response relationships between RT variables such as training duration, frequency, and intensity for T2DM remain under-researched.

**Objectives:**

This meta-analysis aimed to elucidate the overarching effects of RT on HbA1c and FBG metrics and to provide dose-response relationships of RT variables. This was achieved by examining randomized controlled trials (RCTs) that reported reductions in HbA1c and FBG among T2DM patients.

**Methods:**

Comprehensive literature searches were conducted up to 25^th^ February 2023 across databases including EMBASE, Pubmed, Cochrane, CENTRAL, Web of Science, CNKI, Wanfang Data, VIP Database for Chinese Technical Periodicals, and the Chinese Biomedical Database. The Physical Therapy Evidence Database (PEDro) was leveraged to appraise the quality of selected studies based on predefined inclusion and exclusion criteria. The meta-analysis was conducted using Stata 16.

**Results:**

26 studies that include 1336 participants met the criteria for inclusion. RT significantly reduced HbA1c and FBG levels in comparison to control groups (P<0.05). Meta-regression analyses revealed that the number of repetitions per set (p=0.034) was a significant predictor of RT’s efficacy on HbA1c. Subgroup analyses indicated that the most pronounced reductions in HbA1c and FBG occurred with a training duration of 12-16 weeks, intensities of 70-80% of 1 RM, training frequencies of 2-3 times per week, 3 sets per session, 8-10 repetitions per set, and less than a 60-second rest interval.

**Conclusion:**

The beneficial impact of RT on HbA1c and FBG in T2DM patients is affirmed by this systematic review and meta-analysis. Moreover, the critical training parameters identified in this study are pivotal in enhancing HbA1c and FBG reductions, providing a reference for clinical staff to formulate RT exercise regiments for T2DM patients.

**Systematic review registration:**

https://www.crd.york.ac.uk/prospero, identifier CRD42023414616.

## 1 Introduction

Diabetes ranks as the third most prevalent chronic disease globally, trailing only cardiovascular diseases and tumors. An estimated 10.5% of the global population aged between 20 and 79 years suffers from diabetes, and this prevalence is forecasted to escalate to 12.2% (or 783.2 million individuals) by 2045 (1). Notably, T2DM patients constitute over 90% of the overall diabetic population (2). Diabetes can precipitate a myriad of complications, including coronary heart disease, cerebrovascular disease, kidney disease, and blindness, leading to significant health detriments and straining socioeconomic and medical infrastructures (3). In 2021, the global expenditure associated with diabetes-related ailments was pegged at 966 billion USD, with projections indicating an increase to 1054 billion USD by 2045 (1).Exercise elicits numerous metabolic adaptations in the body, prominently including enhanced insulin sensitivity and superior blood glucose regulation (4). Historically, Aerobic training (AT) has been lauded as the quintessential strategy for T2DM management (5). However, a growing body of evidence now underscores the unique advantages of RT in glycemic control (6). A series of investigations (6–8) has accentuated the role of resistance exercise in efficaciously managing type 2 diabetes. The American College of Sports Medicine (ACSM) posits that T2DM patient exercise regimens should incorporate resistance exercises, viewing them as both safe and efficacious modalities (9).

Glycated hemoglobin (HbA1c) serves as an indicator of a patient’s blood glucose trajectory over the preceding two to three months (10). It’s routinely harnessed for diagnosing diabetes, supervising blood glucose consistency, and directing T2DM patient care – gaining esteem as a longstanding “gold standard” for gauging blood glucose oversight (11). Both FBG and HbA1c have been advocated as pivotal markers for diabetic glycemic management (12). Yun et al. (13) deduced from their investigation, which surveyed the interplay between resistance exercise and HbA1c levels among Korean diabetic subjects, that RT augments HbA1c level regulation. A recent meta-analysis (6) corroborated the premise that RT efficaciously diminishes HbA1c in adult T2DM individuals, strongly bolstering reductions in both HbA1c and FBG. Yet, one encompassing meta-analysis comprising 24 trials revealed high-intensity RT to outperform moderate-intensity RT in lowering HbA1c levels within T2DM cohorts (14). Conversely, moderate-intensity RT spanning 12 weeks manifested a palpable drop in FBG for T2DM patients (15). However, high-intensity RT (≥70% 1RM) failed to showcase discernible declines in FBG levels 26 weeks post-training among diabetic individuals (16). Venturing to pinpoint the optimal resistance training regimen, Ishiguro et al. (17) discerned that intervention duration bore no influence on RT’s impact on HbA1c, suggesting potential variability based on specific RT parameters (intensity, frequency, sets). The endeavor to elucidate the ideal quantification of exercise-associated variables during RT for optimal HbA1c reduction and FBG control remains ongoing. Moreover, considering the methodological caveats in extant meta-analyses, which incorporate non-randomized controlled trials (RCTs) (4) and exclusively hinge on direct group comparisons (e.g., high-intensity *vs*. low-intensity) (14), a pressing need emerges to evaluate exercise variables via dose-response relationships rooted in comprehensive systematic reviews and meta-analyses.

Thus, the objective of this meta-analysis was to ascertain the overarching impact of RT on HbA1c and FBG metrics. We employed meta-regression to scrutinize how particular training parameters (e.g., duration, frequency, and intensity) influence HbA1c and FBG values. Furthermore, we delineated dose-response associations for pivotal RT variables by analyzing RCTs that demonstrated reductions in HbA1c and FBG among T2DM patients.

## 2 Methods

In alignment with the Preferred Reporting Items for Systematic Reviews and Meta-Analysis (PRISMA) guidelines (18), we conducted our study. Our research protocol was registered with PROSPERO (CRD42023414616). The PRISMA checklist can be found in the **Supplementary Table S1**.

### 2.1 Strategy of searching

Searches were conducted on databases including EMBASE, PubMed, Web of Science, Cochrane Central Register of Controlled Trials (CENTRAL), Wanfang Data, CNKI, VIP Database for Chinese Technical Periodicals, and the Chinese Biomedical Database. The search spanned until February 25, 2023, guided by predefined search strategies utilizing medical subject headings (MeSH) or their synonyms. We imposed no constraints on publication date, study design, or language. Results were imported into the Endnote bibliographic management tool. The specific search strategies employed in Embase are detailed in the **Supplementary Table S2**.

### 2.2 Selection criteria

The PICOS principle (19) shaped our selection process: (a) Population: Inclusion criteria entailed patients diagnosed with type 2 diabetes aged >18 years. (b) Intervention: RT that detailed at least one training variable (e.g., training intensity). (c) Comparator: Control groups that lacked physical intervention (e.g., health education, no intervention). (d) Outcome: Must report both pre- and post-intervention values for at least HbA1c or FBG. (e) Study Design: RCTs.

Exclusion criteria included: (a) Patients diagnosed with gestational diabetes. (b) Interventions not exclusive to RT (e.g., RT combined with aerobic exercise). (c) Previously published or duplicate literature. (d) Document types like “case reports”, “reviews”, “meta-analysis”, and “letters”.

### 2.3 Data extraction

Two researchers (SW, TK) independently screened titles and abstracts based on our selection criteria, further examining papers that initially met the criteria. Any discrepancies between their selections were addressed through discussion or, if necessary, mediation by a third researcher (ML). Extracted data covered details such as authors, country, sample size, gender, age, body mass index (BMI), disease duration, and RT training variables (e.g., period, intensity, sets, repetitions). RT groups were categorized by training intensity: high-intensity (≥70% 1RM), moderate-intensity (51%-69% 1RM), and low-intensity (≤50% 1RM) (20). After extraction, the two primary researchers cross-checked their findings. In instances where critical data was absent or unclear in the studies, we reached out to the authors for clarification.

### 2.4 Assessment of methodological study quality

Two independent reviewers utilized the Physical Therapy Evidence Database (PEDro) scale (21) to gauge the methodological quality of the studies. The PEDro scale, encompassing 11 criteria, integrates three from the Jadad scale (22) and nine from the Delphi list (23). RCTs are scored between 0-10 (from low to high quality) on the PEDro scale, with scores ≥6 indicating high-quality studies (21). The scale’s first item, specifying eligibility criteria, is geared towards establishing external validity and isn’t factored into the cumulative score. Previous evaluations by Maher et al. (21) confirmed an inter-rater reliability intraclass correlation coefficient of 0.68, based on consensus ratings from two or three independent raters.

### 2.5 Statistical analyses

To evaluate the overall effect of RT on HbA1c and FBG and establish a dose-response relationship in patients with type 2 diabetes, the standardized mean differences (SMD) was calculated using the formula: formula: SMDi = $\frac{m1i-m2i}{Si}$ (24). Here, SMD*i* represents the standardized difference between reported measures, where m1*i* and m2*i* represent the means of the intervention and control groups, respectively, while s*i* is the pooled standard deviation. This formula was adjusted for sample size by Hedges and Olkin as: g =( 1- $\frac{3}{4Ni-9}$ ) (24), where *N_i_* denotes the combined sample size of both groups. SMD signifies the difference between post-test averages of the treatment and control groups divided by the combined group’s standard deviation and is accompanied by 95% confidence intervals (CIs). Due to significant heterogeneity among studies, stemming from factors such as varying muscle groups, a random-effects model was utilized to estimate the effect of RT interventions (19). Our categorical variable meta-analysis was executed using Stata 16 software. Cohen categorized effect sizes as: 0.00 to ≤0.49 for small, 0.50 to ≤0.79 for moderate, and ≥0.80 for large effects (25). For clarity, a positive SMD was reported when RT exhibited superiority over the control group. The I² statistic assessed heterogeneity. Additionally, meta-analytic regression (metareg) investigated if the combined values of diverse training variables could predict RT’s influence on HbA1c and FBG. Subcategories were established to identify pivotal training variables, encompassing training duration, frequency, repetitions per session, repetitions per set, intensity, and rest intervals. Each subcategory was examined using a random-effects meta-regression model to discern potential enhancements in HbA1c and FBG measurements.

## 3 Results

**Figure 1:**
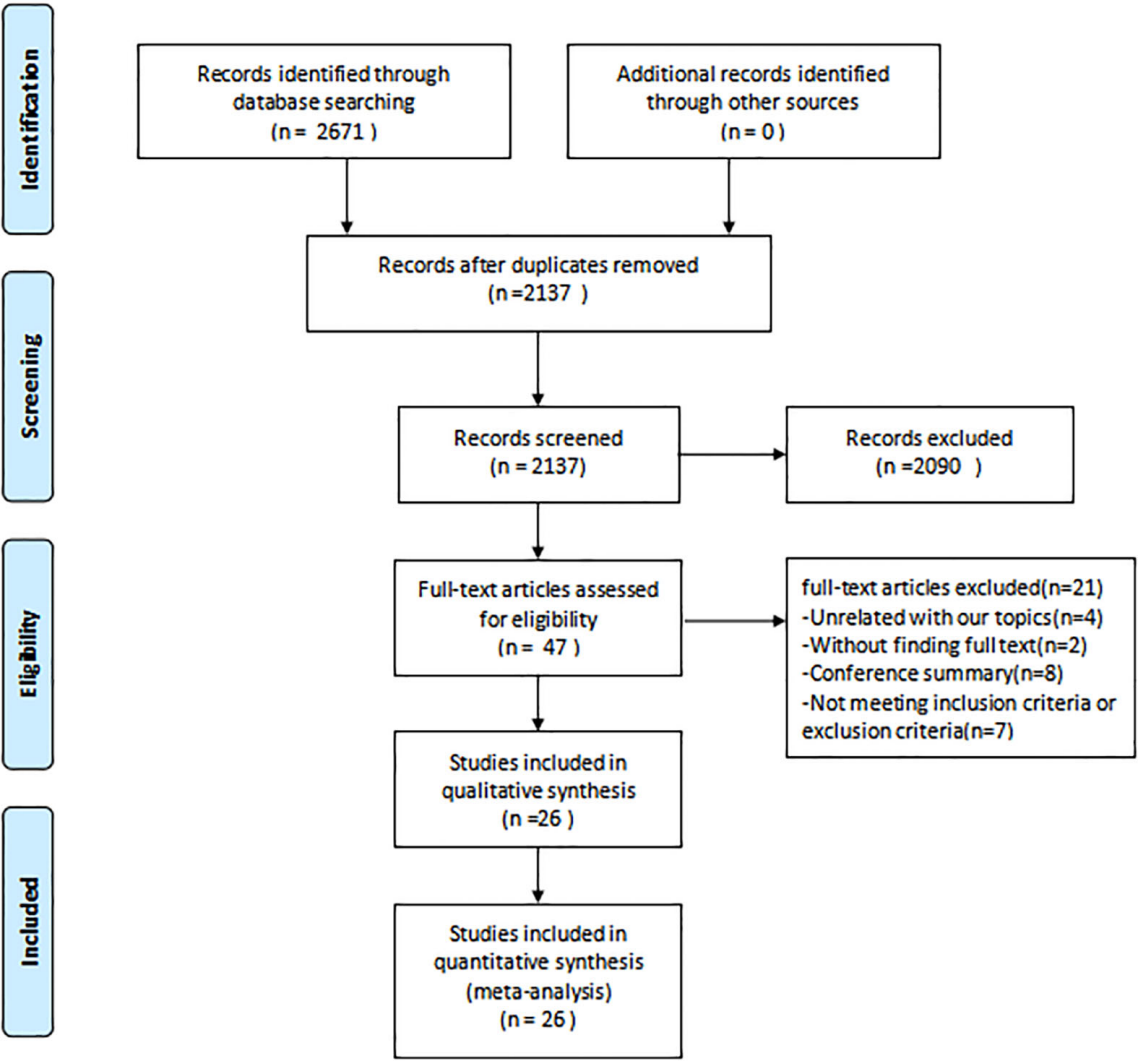
Flowchart of the selection of studies included in the meta-analysis.

**Figure 1** depicts the PRISMA flowchart detailing the literature search process. An initial search yielded 2671 potential studies. After the exclusion of duplicates, 2137 studies were retained. A screening based on titles and abstracts resulted in the removal of 2090 studies. Of the 47 articles that remained, 21 were excluded due to irrelevance to the subject, absence of full text, or unavailability of conference abstracts. Thus, the quantitative synthesis incorporated 26 studies (15, 16, 26–49) comprising 1336 participants; the characteristics of these studies are presented in **Table 1**. The intervention groups in these studies had sample sizes ranging from 9 to 165 participants, with ages spanning 47 to 73 years and diabetes duration from 1 to 18 years. RT interventions lasted between 8 and 52 weeks, with training frequencies of 1 to 7 sessions weekly, encompassing 1-5 sets per session, 8-20 repetitions per set, and rest intervals of 30-600 seconds between sets. The reviewed literature indicated training intensities between 30% and 80% of a single repetition maximum (RM). Concerning RT modalities, 8 studies utilized body weight, 1 employed machines, 7 combined machines and body weight, 1 used elastic bands, 5 incorporated both elastic bands and body weight, 1 employed body weight and sandbags, 1 integrated machines, elastic bands, and body weight, 1 combined body weight, machines, and dumbbells, while 2 studies implemented a complex training approach.

**Table 1:** Characteristics of the trials included in the meta-analysis.

Study, Year	Country	N	Sex(M/F)	Age(Y)	BMI (kg/m2)	Diabetes Duration (Y)	RT exercise	TP	TF	TI	Sets	Reps	Rest
Arora et al. (26), 2009	India	C=10 I=10	C=6/4 I=4/6	C=58.4±1.8 I=49.6±5.2	C=24.98±3 I=26.99±4.1	C=5.2±2.9 I=5.4±1.5	RT base on self-weight, Dumbbells, pulleys, lateral pull down and quadriceps table.	8	2	60–100	3	10	300
Baldi et al. (27), 2003	New Zealand	C=9 I=9	NR	C=50.1±1.3 I=46.5±2.1	C=36.4±3.1 I=34.3±3.2	> 3	RT base on self-weight	10	3	65–75	2	12	NR
Books et al. (28), 2007	America	C=31 I=31	C=19/12 I=21/10	C=66 ± 1 I=66 ± 2	C=31.2 ± 1.0 I=30.9 ± 1.1	C=11±1 I=8±1	RT base on self-weight, machines	16	3	60–80	3	8	300
Castaneda et al. (29), 2002	America	C=31 I=31	C=19/21 I=21/10	C=66±1 I=66±2	C=31.2±1.0 I=30.9±1.1	C=11±1 I=8±1	RT base on self-weight, machines	14	3	60-80	3	8	NR
Chen et al (30), 2019	Taiwan	C=30 I=30	C=18/12 I=11/19	C= 65.0±3.1 I= 65.9 ±2.9	C=28.1±4.9 I=26.7±4.2	C=11.5±7.8 I=9.1±7.4	RT base on elastic band	12	3	NR	5	10	NR
Cheung et al. (31), 2009	Ireland	C=17 I=20	C=12/5 I=13/7	C=62±6.7 I=59±8.7	C=37.7±9.0 I=39.7±9.0	NR	RT base on self-weight, exercise bands	16	5	NR	2	12	300
Chien et al. (32), 2022	China	C=20 I=20	C=5/15 I=2/18	C=67.3±6.1 I=67.6±7.7	C=25.5±3.7 I=24.3±3.4	C=13.6±7.6 I=17.5±16.3	RT base on self-weight, sandbag	12	3	NR	3	20	60-180
Church et al. (33), 2010	America	C=41 I=73	C=13/28 I=30/43	C=55.8±8.7 I=55.8±8.7	C=34.8±6.2 I=34.1±5.4	C=7.2±5.2 I=7.2±5.5	RT base on self-weight	36	3	67	2–3	10–12	NR
De Oliveira et al. (34), 2012	Brazil	C=12 I=10	C=4/8 I=4/6	C=53.4±9.8 I=54.1±8.9	C=30.0±4.9 I=31.3±4.1	C=5.3±3.5 I=7.7±3.9	RT base on self-weight	12	3	50	2–4	8–12	2
Dunstan et al. (35), 1998	Australia	C=10 I=11	C=5/5 I=8/3	C=51.1±2.2 I=50.3±2.0	C=30.1±1.1 I=28.3±0.8	C=5.1±1.2 I=5.3±1.4	RT base on self-weight	8	3	50–75	3	10–15	60
Giessing et al. (16), 2022	Germany	C=28 I=29	40/17	62±3.9	31.8±1.4	NR	RT base machines	26	2	>75	NR	8-10	10-29
Gordon et al. (36), 2006	America	C=15 I=15	C=8/7 I=7/8	C=67±2 I=67±2	C=33.5±1.6 I=30.7±1.6	C=12±3 I=9±2	RT base on self-weight, Keiser pneumatic variable resistance equipment.	16	3	60–80	3	8	60-120
Hangping et al. (37), 2019	China	C=100 I=165	C=43/57 I=78/87	C=66.7±6.7 I=65.7±8.7	C=25.3±3.3 I=25.04±3.32	C=8± 2.4 I=9±2.4	RT base on machines	24	1	>75	4	NR	NR
Ku et al al. (38), 2010	Korea	C=16 I=13	C=0/16 I=0/13	C=57.8±8.1 I=55.7±6.2	C=27.4±2.8 I=27.1±2.3	C=5.8±6.1 I=5.7±4.8	RT base on self-weight, Elastic bands	12	5	40–50	3	15–20	NR
Kwon et al. (39), 2011	Korea	C=15 I=12	C=0/15 I=0/12	C=58.9±5.7 I=56.3±6.1	C=27.0±2.3 I=27.4±2.1	C=4.9±4.7 I=4.6±2.7	RT base on self-weight, Resistance bands	12	3	40–50	3	10–15	600
Mohammadi (40), et al. 2022	Iran	C=12 I=12	NR	NR	C=28.8±2.3 I=28.8±2.1	NR	RT base on self-weight	12	3	68	3	12	90
Nadi et al. (15), 2019	Iran	C=15 I=15	C=0/15 I=0/15	C=54.8±3.3 I=56.1±3.4	C=27.2±2.6 I=30.41±0.12	C=11.2±3.2 I=11.2±1.9	RT base on self-weight	12	3	30	NR	9	30
Plotnikoff et al. (42), 2010	Canada	C=21 I=27	C=8/13 I=8/19	C=54±12 I=55±12	C=36.0±5.0 I=35.0±8.0	NR	RT base on self-weight, Weight machines dumbbells	16	3	50–85	2–3	8–12	90-120
Ranasinghe et al. (43), 2021	Australia	C=30 I=28	C=16/14 I= 13/15	C= 49.3±7.0 I=49.0 ±9.2	NR	NR	RT base on self-weight, machines	12	2	50-60	3	8–10	NR
Rezaeeshirazi (44), 2022	Iran	C=15 I=14	C=15/0 I= 14/0	NR	C=32.5±1.5 I=32.0±1.3	NR	RT base on machines	8	4	50-70	3	8-15	50-70
Shenoy et al. (45), 2009	India	C=10 I=10	C=6/4 I=4/6	C=58.4±1.8 I=49.6±5.2	C=25.0 ±3.1 I=26.9±4.1	C=5.2±2.9 I=5.4±1.5	RT base on self-weight, quadriceps table	16	2	60–100	3	10	NR
Sigal et al (46), 2007	Canada	C=63 I=64	C=41/22 I=40/24	C=54.8±7.2 I=54.7±7.5	NR	C=5.0±4.5 I=6.1±4.7	RT base on self-weight, machines	26	3	80	2–3	7–9	NR
Sparks et al (41), 2013	America	C=10 I=18	C=23/29 I=23/29	C=57.6±7.5 I=57.6±7.5	C=36.4±4.0 I=33.9±5.2	C=5.4±3.3 I=9.4±6.8	RT base on self-weight	39	3	NR	3	10–12	NR
Wycherley et al (47), 2010	Australia	C=16 I=17	NR	C=55.0±8.4 I=55.0±8.4	C=35.3±4.5 I=35.3±4.5	NR	RT base on self-weight	16	3	70–85	2	8–12	60-120
Yamamoto et al (48), 2021	Japan	C=17 I=18	C=10/7 I=9/9	C=73.3 ±2.5 I=73.2 ± 2.6	C=24.4 ± 4.7 I=23.8 ± 3.0	C=17.3 ± 9.6 I=17.6 ± 10.0	RT base on self-weight, Elastic bands	48	7	NR	1	20	NR
Yavari et al (49), 2012	Iran	C=20 I=20	NR	C=51.5±8.5 I=51.5±6.3	NR	NR	RT base on self-weigh, machines	52	3	75–80	3	8–10	1.5-2

I, intervention group; C, control group; N, number of participants; M, male; F, female; NA, not applicable; RT, resistance training; TP, training periods (weeks); TF, training frequency (times per week); TI, training intensity (% of 1 repetition maximum); Sets, number of sets per exercise; Reps, number of repetitions per set; Rest, time of rest between sets (seconds).

### 3.1 Methodological quality of the study

As presented in **Table 2**, the studies exhibited an average quality score of 5.23 ± 1.31 points, with scores ranging from 3 to 8. Notably, six studies (29, 30, 40, 42, 43, 46) secured a score of 6 or above.

**Table 2:** Physiotherapy Evidence Database (PEDro) scores of the 26 included studies.

Authors	Eligibility criteria	Random allocation	Concealed allocation	Baseline comparability	Blind subjects	Blind therapists	Blind assessor	Adequate follow-up dropout\15 %	Intention-to treat analysis	Between-group comparisons	Point estimates and variability	Score
Arora et al. (26), 2009	+	+	–	+	–	–	–	+	–	+	+	5
Baldi et al (27), 2003	–	+	–	–	–	–	–	+	–	+	+	4
Books et al. (28), 2007	–	+	–	+	–	–	–	+	–	+	+	5
Castaneda et al. (29), 2002	+	+	–	+	+	–	–	+	–	+	+	6
Chen et al. (30), 2019	+	+	+	+	+	–	–	+	+	+	+	8
Cheung et al. (31), 2009	+	+	–	+	–	–	–	+	–	+	+	5
Chien et al. (32), 2022	+	+	–	+	–	–	–	+	–	+	+	5
Church et al. (33), 2010	–	+	–	–	–	–	–	–	–	–	+	2
De Oliveira et al. (34), 2012	+	+	–	+	–	–	–	+	–	+	+	5
Dunstan et al. (35), 1998	–	+	–	–	–	–	–	+	–	+	+	4
Giessing et al. (16), 2022	–	+	–	+	–	–	–	+	–	+	+	5
Gordon et al. (36), 2006	+	+	–	+	–	–	–	+	–	+	+	5
Hangping et al. (37), 2019	+	+	–	+	–	–	–	+	–	+	+	5
Ku et al al. (38), 2010	+	+	–	–	–	–	–	+	–	+	+	4
Kwon et al. (39), 2011	+	+	–	+	–	–	–	+	–	+	+	5
Mohammadi (40), et al., 2022	+	+	–	+	–	–	–	+	+	+	+	6
Nadi et al. (15), 2019	+	+	–	+	–	–	–	+	–	+	+	5
Plotnikoff et al. (42), 2010	+	+	–	+	–	–	–	+	+	+	+	6
Ranasinghe et al. (43), 2021	+	+	–	+	–	–	+	+	+	+	+	7
Rezaeeshirazi (44), 2022	+	+	–	+	–	–	–	+	–	+	+	5
Shenoy et al. (45), 2009	+	+	–	+	–	–	–	+	–	+	+	5
Sigal et al. (46), 2007	+	+	+	+	–	+	–	+	+	+	+	8
Sparks et al. (41), 2013	+	+	–	+	–	–	–	+	–	+	+	5
Wycherley et al. (47), 2010	+	+	–	+	–	–	–	–	–	+	+	4
Yamamoto et al. (48), 2021	+	+	–	+	–	–	–	+	–	+	+	5
Yavari et al. (49), 2012	+	+	–	+	–	–	–	+	–	+	+	5

“+” indicates a “yes’’ score, ‘‘-’’indicates a ‘‘no’’ score.

### 3.2 Overall findings

**Figure 2:**
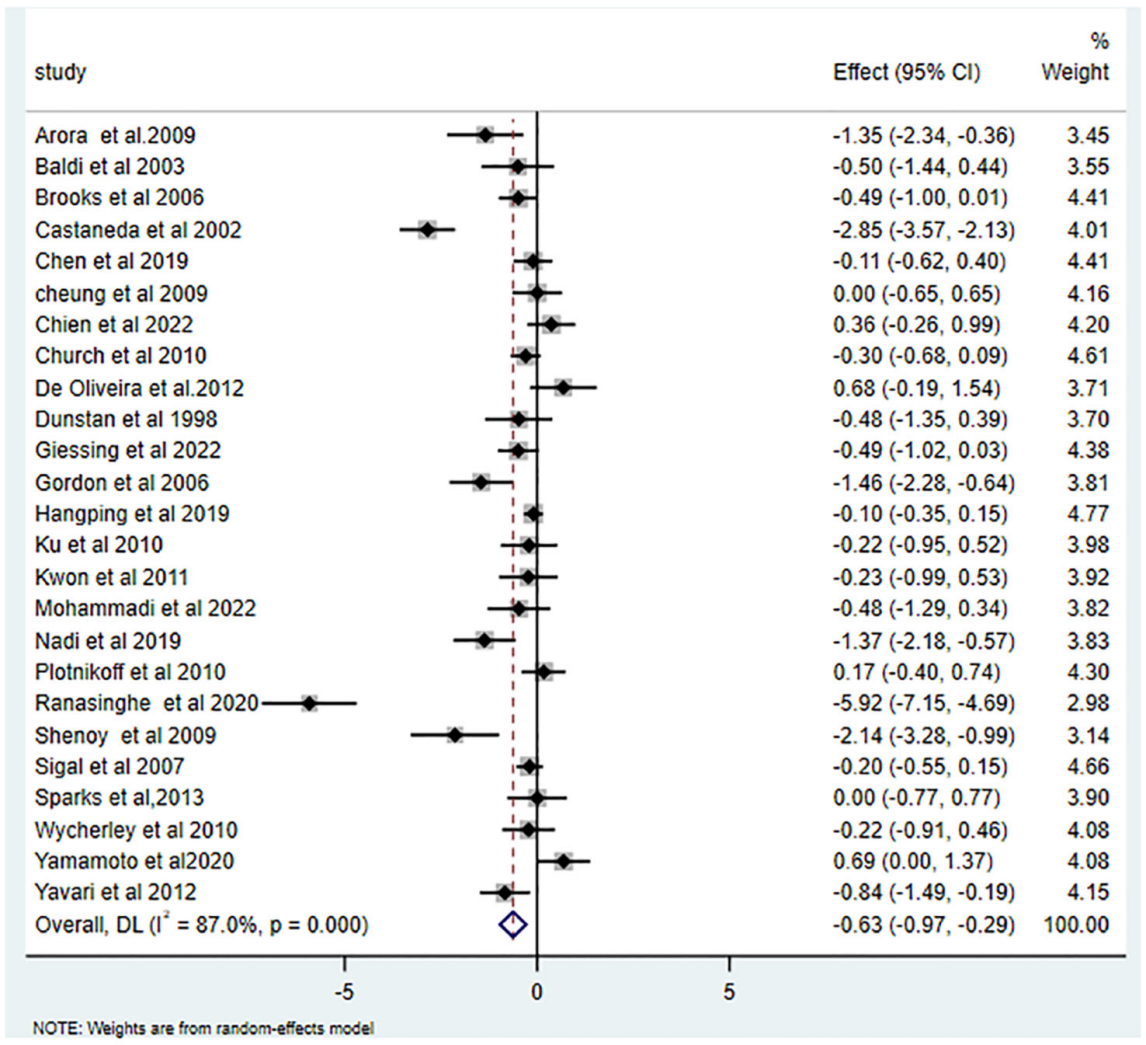
Effects of RT on measures of HbA1c.

**Figure 3:**
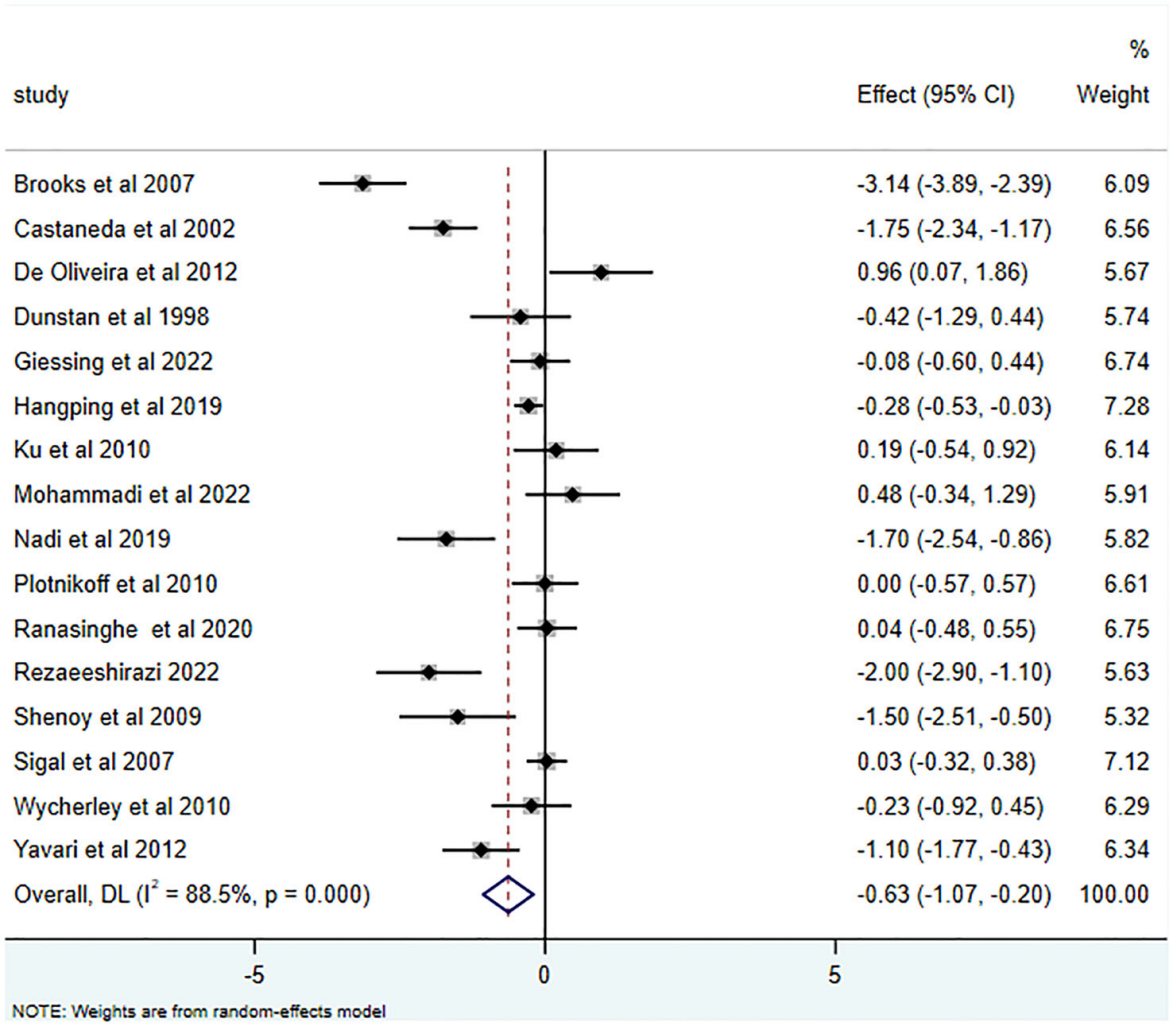
Effects of RT on measures of FBG.

25 studies (15, 16, 26–43, 45–49) reported the effects of RT on HbA1c, yielding an SMD of -0.63 (95% CI: -0.97 to -0.29; I^2 = ^87.0%, p<0.001). Relative to the control group, RT substantially decreased HbA1c levels in T2DM patients, as illustrated in **Figure 2**. Of the 16 studies (15, 16, 28, 29, 34, 35, 37, 38, 40, 42–47, 49) that examined RT’s influence on FBG, the average SMD was -0.63 (95% CI: -1.07 to -0.20; I^2^ = 88.5%, p<0.001). This is depicted in **Figure 3** and highlights the significant effect.

### 3.3 Meta-regression analysis of training variables

Meta-regression results pinpointed the number of repetitions per set (p=0.034) as a predictor of RT’s impact on HbA1c (refer to **Table 3**). Regression analysis, however, revealed that no specific training parameters—including duration, intensity, frequency, number of sets, and number of repetitions—significantly affected FBG measurements (see **Table 4**).

**Table 3:** Meta-regression for training variables to predict RT effects on HbA1c.

	Coefficient	Standard error	95 % lower CI	95 % upper CI	Z value	P
Training period	0.0236664	0.0215485	-0.0209102	0.068243	1.10	0.283
Training intensity	0 .0048011	0.0232869	-0.044123	.0537251	0.21	0.839
Training frequency	0.4149407	0 .2309484	-0.0628125	0.8926938	1.80	0.086
Number of sets	-0.0681706	0.3324552	-0.759549	0.6232079	-0.21	0.840
Number of repetitions per set	0 .1745202	0.0771692	0.0140381	0.3350023	2.26	**0.034**
Rest in between sets	0.0003368	0.0011423	-0.0021521	0.0028257	0.29	0.773

CI, confifidence interval; RT, resistance training. The bold values denote a positive correlation between the number of repetitions per set and HbA1c.

**Table 4:** Meta-regression for training variables to predict RT effects on FBS.

	Coefficient	Standard error	95 % lower CI	95 % upper CI	Z value	P
Training period	-0.0012369	0.0262082	-0.0574478	0.0549741	-0.05	0.963
Training intensity	-0.0030403	0.0200405	-0.046023	0.0399423	-0.15	0.882
Training frequency	-0.3519978	0.3955662	-1.200403	0.4964072	-0.89	0.389
Number of sets	0.4008127	0.4338931	-0.5445591	1.346185	0.92	0.374
Number of repetitions per set	0.1600267	0.1336572	-0.1311874	0.4512408	1.20	0.254
Rest in between sets	-0.007458	0.004809	-0.0185476	0.0036316	-1.55	0.160

CI, confifidence interval; RT, resistance training.

### 3.4 Subcategories analysis of training variables

#### 3.4.1 Subcategories analysis of training vtariables on HbA1c

Effect sizes for each training parameter were independently computed to discern their influence on HbA1c (as detailed in **Table 5**). Analysis inferred that a training duration of 12-16 weeks, intensities equating to 70-80% of 1RM, 2-3 weekly training sessions, 3 sets per activity, 8 to 10 repetitions within each set, and inter-set rest periods under 60 seconds yield the optimal reduction in HbA1c.

**Table 5:** Training variables with Subcategories in HbA1c.

Training variables	Effect	95 % lower CI	95 % upper CI	Z value	p
Training period [weeks]	-0.63	-0.97	-0.29	-3.632	0.000
≥8,<12	-0.39	-1.21	0.42	-0.943	0.346
≥12,<16	-1.29	-2.38	-0.20	-2.320	0.020
≥16,<24	-0.58	-1.16	-0.01	-1.996	0.046
≥24	-0.20	-0.46	0.06	-1.538	0.124
Training intensity [% of 1RM]	-0.85	-1.25	-0.45	-4.162	0.000
≤50%	-0.29	-1.08	0.49	-0.735	0.462
≥51%,≤69%	-1.30	-2.67	0.07	-1.866	0.062
≥70%	-0.89	-1.35	-0.44	-3.824	0.000
Training frequency [sessions per week]	-0.63	-0.97	-0.29	-3.632	0.000
1	-0.1^a^	-0.35	0.15	-0.768	0.442
2	-2.43	-4.58	-0.29	-2.221	0.026
3	-0.46	-0.78	-0.14	-2.801	0.005
>3	0.33^a^	-0.34	1.01	0.973	0.331
Number of sets per exercise	-0.61	-0.97	-0.24	-3.288	0.001
1	0.69^a^	0.00	1.37	–	–
2	-0.18	-0.61	0.24	-0.861	0.389
3	-0.92	-1.49	-0.36	-3.288	0.001
>3	-0.13	-0.34	0.07	-1.264	0.206
Number of repetitions [per set]	-0.65	-1.05	-0.24	-3.121	0.002
6-10	-1.31	-2.03	-0.58	-3.548	0.000
≥11,≤15	-0.19	-0.43	0.04	-1.590	0.112
≥16	0.30	-0.20	0.79	1.176	0.240
Rest in between sets [s]	-0.41	-0.75	-0.07	-2.365	0.018
≤60	-0.94^a^	-1.82	-0.06	-2.104	0.035
>60,≤120	-0.25	-0.76	0.26	-0.948	0.343
>120	-0.43	-0.90	0.03	-1.840	0.066

The content of this table is based on individual training variables with no respect for interaction between training variables SMD between-subject standardized mean difference, 1RM one-repetition maximum, ^a^ Based on less than three studies.

#### 3.4.2 Subcategories analysis for training variables on FBG

Effect sizes for each training parameter, in the context of FBG, were separately evaluated (refer to **Table 6**). It emerged that 70-80% of 1RM intensities, 3 weekly training sessions, 3 sets per session, 8 to 10 repetitions per set, and rest intervals shorter than 60 seconds between sets are most conducive to FBG reduction.

**Table 6:** Training variables with Subcategories in FBS.

Training variables	Effect	95 % lower CI	95 % upper CI	Z value	p
Training period [weeks]	-0.63	-1.07	-0.20	-2.871	0.004
≥8,<12	-1.21^a^	-2.75	0.34	-1.529	0.126
≥12,<16	-0.31	-1.20	-0.58	-0.681	0.496
≥16,<24	-1.20	-2.62	-0.26	-1.609	0.108
≥24	-0.29	-0.65	0.07	-1.563	0.118
Training intensity [% of 1RM]	-0.63	-1.07	-0.20	-2.871	0.004
≤50%	-0.18	-1.67	1.31	-0.241	0.810
≥51%,≤69%	-0.34	-1.03	0.36	-0.953	0.341
≥70%	-0.96	-1.59	-0.33	-2.987	0.003
Training frequency [sessions per week]	-0.63	-1.07	-0.20	-2.871	0.004
1	-0.28^a^	-0.53	-0.03	–	–
2	-0.39	-1.10	-0.33	-1.057	0.291
3	-0.61	-1.26	0.04	-1.846	0.065
>3	-2.00^a^	-2.90	-1.10	–	–
Number of sets per exercise	-0.61	-1.08	-0.13	-2.511	0.012
1	–	–	–	–	–
2	-0.23^a^	-0.92	0.45	-0.669	0.503
3	-0.87	-1.57	-0.17	-2.442	0.015
>3	0.00	-0.47	0.48	0.013	0.990
Number of repetitions [per set]	-0.64	-1.21	-0.07	-2.185	0.029
6-10	-0.73	-1.45	-0.02	-2.008	0.045
≥11,≤15	-0.64	-2.05	0.77	-0.887	0.375
≥16	0.19^a^	-0.54	0.92	0.508	0.611
Rest in between sets [s]	-0.79	-1.62	0.03	-1.893	0.058
≤60	-1.07	-1.95	-0.19	-2.375	0.018
>60,≤120	-0.05	-0.79	0.89	0.118	0.906
>120	-3.14^a^	-3.89	-2.39	–	–

The content of this table is based on individual training variables with no respect for interaction between training variables SMD between-subject standardized mean difference, 1RM one-repetition maximum, ^a^ Based on less than three studies.

## 4 Discussion

In this study, we examined the impact of RT on HbA1c and FBG levels in T2DM patients. Our meta-regression sought to identify the most influential training variables on these outcomes after RT. We further analyzed the dose-response relationships for each training variable. Key findings include: (1). RT effectively decreases both HbA1c and FBG in T2DM patients. (2). The number of repetitions per set emerged as a predictor of RT’s effect on HbA1c. (3). The optimal RT regimen to ameliorate HbA1c and FBG in T2DM patients encompasses a training duration of 12-16 weeks, an intensity of 70-80% of 1 RM, frequency of 2-3 times per week, and performing 3 sets of 8-10 repetitions for each exercise with less than a 60-second rest interval.

### 4.1 RT’s effect on HbA1c and FBG in T2DM patients

Our data suggests a substantial benefit of RT on HbA1c levels in T2DM patients (-0.63 (95% CI -0.97- -0.29; I^2^ = 87.0%, p=0.000)), aligning with previous studies (14, 17, 50). Effective HbA1c management can attenuate diabetes-related complications and mortality (51). Maintaining glucose levels at 7% or below can curtail long-term complications by up to 76% (49). A 1% decline in HbA1c can potentially decrease diabetes-related myocardial infarction risk by 14% and overall mortality by 21% (52). Our results are in line with Kelly, who reported an approximate 0.8% decrease in HbA1c through exercise, optimizing glycemic control (53). The therapeutic potential of RT may arise from augmented glucose transporter presence, increased muscle mass, and enhanced insulin receptor activity in muscle cells (54).

Furthermore, RT has been acknowledged for its efficacy in diminishing FBG in T2DM patients (-0.63 (95% CI -1.07- -0.20; I^2^ = 88.5%, p=0.000)). This is consistent with Yaping et al., who posited that RT exerts a more potent influence on FBG enhancement (55). Brooks et al. (28), Castaneda et al. (29), and Yavari et al. (49) reported analogous findings. Mechanisms underlying these improvements include increased muscle strength, augmented GLUT-4 receptor activity, rapid glucose transportation, and refined insulin resistance and sensitivity (56). Rezaeeshirazi et al. (44) also reported increased insulin sensitivity in elderly T2DM patients’ post-resistance exercise, leading to substantial FBG reduction.

### 4.2 Dose-response correlations of RT to reduce HbA1c

Numerous studies have established that various training variables, such as duration, frequency, and intensity, within sports training can optimize the hypoglycemic effect (14, 57). Consequently, discerning which variable correlates with a more potent positive impact of exercise on T2DM patients becomes crucial. Our analysis, encompassing 25 studies (15, 16, 26–43, 45–49), revealed that the duration of RT had no significant association with HbA1c reduction. Training durations ranging from 12-16 weeks seem to be the most effective in decreasing HbA1c. This may be attributed to the notion that a noticeable exercise effect necessitates a more extended training duration. For instance, Dunstan et al. (35) observed no marked improvement in HbA1c following 8 weeks of moderate-intensity RT. The lack of significant change in HbA1c could be due to brief training durations, specifically, 4-6 weeks (58). Conversely, even with prolonged RT cycles, some patients might not achieve the desired results, possibly due to inadequate exercise intensity or compliance. Church et al. (33) found no significant amelioration in HbA1c after 36 weeks of moderate-intensity RT, and Yamamoto et al. (48) reported 27 participants dropping out of their study. Hence, intervention duration alone doesn’t dictate HbA1c reduction but is influenced by both training intensity and adherence.

Our meta-analysis, which incorporated 20 studies (15, 16, 26–29, 33–40, 42, 43, 45–47, 49), recorded a 0.89% and 0.29% decrease in HbA1c with high and low intensity RT, respectively. This aligns with another meta-analysis (encompassing 24 trials) which suggested that high-intensity RT is superior in reducing HbA1c in T2DM patients as compared to low-to-moderate intensity RT (48). Contrarily, a separate Australian study observed that 3 months of moderate-intensity RT led to a significant decline in HbA1c levels (by 5.92%) in T2DM patients (43). In our research, it was evident that when the mean baseline HbA1c exceeded 7.5%, participants displayed greater HbA1c improvement post-RT, a finding corroborated by studies from Segal et al. (Canada) (46) and Kadoglou et al. (Greece) (59). However, the intensity of RT didn’t exhibit a direct correlation with its effect on HbA1c reduction in diabetic patients.

Moreover, our analysis indicated that conducting 2-3 sets per week and 3 sets per exercise session were most effective in HbA1c reduction. Conventionally, the advised frequency for RT is two to three times weekly, although it’s recommended to be integrated with AT (60). Excessive training frequency could lead to fatigue; hence, the number of sets might not be the primary determinant for HbA1c reduction in T2DM patients. Meta-regression analysis underscored the pivotal role of repetition count in HbA1c decline. T2DM patients witnessed the most significant drop in HbA1c with 6-10 repetitions per set (mean SMD= 1.31). While the aggregate repetitions in an RT set might induce a substantial physiological stimulus for enhanced strength and subsequent HbA1c reduction, fewer repetitions might be more potent (17). This is because repetition count is often equated with training intensity. Historically, emphasis was on “training intensity” rather than “number of repetitions”, leading to the inference that fewer repetitions correspond to augmented training intensity (61). Lastly, no discernible correlation existed between RT rest periods and HbA1c reduction. Rest intervals under 60 seconds appeared most effective in diminishing HbA1c in our study. A potential rationale is that shorter rest intervals induce more fatigue, thus offering a stimulus augmenting muscular strength and subsequently lowering HbA1c (62).

### 4.3 Dose-response correlations of RT to reduce FBG

Sixteen studies (15, 16, 28, 29, 34, 35, 37, 38, 40, 42–47, 49) were incorporated into this analysis. Meta-regression identified no significant association between training variables and FBG. Nonetheless, the optimal RT program for enhancing FBG in T2DM patients is characterized by a training intensity of 70-80% of 1RM, conducted thrice weekly, encompassing 3 sets during each session, with 8-10 repetitions, and inter-set rest intervals of less than 60 seconds.

Evidence from randomized controlled trials indicates distinct glycemic responses in T2DM patients undergoing RT. Notably, a 26-week high-intensity RT (70% 1RM) regimen yielded minimal alterations in blood glucose levels (16). Conversely, a 12-week moderate-intensity RT regimen precipitated notable reductions in fasting blood glucose (15). However, a separate 12-week moderate-intensity RT program (50-60% 1RM) failed to elicit significant glucose reductions (43). A recent meta-analysis posited that older T2DM patients should prioritize RT intensity over duration and frequency to ensure optimal glycemic management (50). Collectively, the impact of RT on glycemic fluctuations appears to be heterogenous, and the standalone effect of RT intensity on glucose levels in diabetics remains nuanced.

Subgroup analysis from our research reveals that a training intensity of 70-80% of 1RM, executed thrice weekly in 3 sets, with 8-10 repetitions per set, and rest periods of less than 60 seconds between sets, is most conducive for FBG reduction. These observations align with prior studies (28, 29, 47). Ferriolli et al. (63) suggested an RT protocol for elderly diabetic patients, advocating for sessions on at least 2 days per week at a moderate-to-high intensity of 1RM. Similarly, Liu et al.’s meta-analysis (14) endorsed an RT regimen with an average high intensity (75-100% of 1RM), performed in 2-3 sets per week and 8-10 repetitions per set, as efficacious in reducing FBG among T2DM patients. Furthermore, in scenarios devoid of contraindications, Pan et al. (64) recommended diabetic patients to train thrice weekly, targeting all major muscle groups over 3 sets of 8-10 repetitions, but never exceeding 10 repetitions. These recommendations resonate with the insights of our meta-analysis. Despite these insights, it remains imperative to further probe the efficacy of resistance exercise interventions on FBG.

### 4.4 Limitations of the meta-analysis

A notable limitation of our study is the inability to conduct a thorough analysis of the interrelationships among the suggested training variables. Our research draws from several studies that employed diverse training parameter combinations, such as intensity, training frequency, and number of sets. Given the variability in these parameters, it remains inconclusive whether the outcomes would remain optimal if each element of the RT method were tailored based on the prevailing dose-response relationship. Hence, further investigations are imperative to devise an analytical framework that provides insights into the interplay between these training parameters. One approach to overcome this limitation is through the systematic modeling of training variables, wherein one RT variable remains constant while another is varied. While our study evaluated the influence of radiation on HbA1c and FBG, the assessment did not extend to hypertrophy (specifically, alterations in lean tissue) due to the paucity of studies documenting such changes. Consequently, the direct link between exercise and augmented muscle mass remains speculative. We advocate for future studies examining the relationship between hypertrophy and HbA1c.

Moreover, the majority of the studies reviewed did not provide details on medication adjustments throughout the intervention. If the control group received a more potent medication regimen compared to the intervention group, the impact of RT on glycemic control could have been underestimated. This was particularly evident in studies with combined medication interventions. Another challenge encountered in our literature review and meta-analysis was the pronounced bias inclination of the included studies (only 6 out of 26 studies attained a PEDro score of 6), the marked heterogeneity across the studies (i.e., I^2 values ranging from 59.5% to 88.5%), and the uneven SMD reported for specific training parameters.

## 5 Conclusion

This literature review and meta-analysis substantiate that RT effectively reduces HbA1c and FBG levels in T2DM patients. An optimal training regimen encompasses a duration of 12-16 weeks, an intensity set at 70-80% of 1 RM, a frequency of 2-3 sessions per week, and a structure of 3 sets with 8-10 repetitions for each exercise. Moreover, allowing a rest interval of less than 60 seconds between sets has been demonstrated to be efficacious in lowering HbA1c and FBG levels. Such a regimen can provide reference for clinical researchers to develop exercise training programs tailored for T2DM patients.

## Data availability statement

The original contributions presented in the study are included in the article/**Supplementary Material**, further inquiries can be directed to the corresponding author/s.

## Author contributions

WS: Conceptualization, data curation, formal analysis, methodology, software, writing original draft, writing review and editing, and translate. KT, FX, and MT: translate, validation, supervision, writing review and editing, data curation, and methodology. LM and XD: validation, software, and investigation. YQ: conceptualization, project administration, resources, validation, supervision, and writing review and editing. All authors contributed to the article and approved the submitted version.
