# Supplementary material for: Dose-response relationships of resistance training in Type 2 diabetes mellitus: a meta-analysis of randomized controlled trials
Source: Front Endocrinol (Lausanne). 2023 Sep 25;14:1224161. doi: 10.3389/fendo.2023.1224161 (PMC10561623; doi:10.3389/fendo.2023.1224161)
Supplement: Supplementary file 1 [file Table_1.docx]

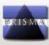
**PRISMA** **2020** **Checklist**

| **Section** **and** **Topic** | **Ite**  **m** **#** | **Checklist** **item** | **Location** **where** **item** **is** **reported** |
| --- | --- | --- | --- |
| **TITLE** | | |  |
| Title | 1 | The report is identified a meta-analysis. |  |
| **ABSTRACT** | | |  |
| Abstract | 2 | The structured abstract includes background, Methods, Results and Conclusions. |  |
| **INTRODUCTION** | | |  |
| Rationale | 3 | Described in the introduction. |  |
| Objectives | 4 | Described in the introduction. |  |
| **METHODS** | | |  |
| Eligibility criteria | 5 | Described in the methods. |  |
| Information sources | 6 | Described in the methods. | Supplementary Table S1 |
| Search strategy | 7 | Described in the methods. | Supplementary Table S2 |
| Selection process | 8 | Described in the methods. |  |
| Data collection  process | 9 | Described in the methods. |  |
| Data items | 10a | Described in the methods. |  |
|  | 10b | Described in the methods. |  |
| Study risk of bias  assessment | 1 1 | Described in the methods. |  |
| Effect measures | 12 | Described in the methods. |  |
| Synthesis methods | 13a | Described in the methods. |  |
|  | 13b | Described in the methods. |  |
|  | 13c | Described in the methods. |  |
|  | 13d | Described in the methods. |  |
|  | 13e | Described in the methods. |  |
|  | 13f | Described in the methods. |  |
| Reporting bias  assessment | 14 | Described in the methods. |  |
| Certainty assessment | 15 | Described in the methods. |  |


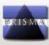
**PRISMA** **2020** **Checklist**

| **Section** **and** **Topic** | **Ite**  **m** **#** | **Checklist** **item** | **Location** **where** **item** **is** **reported** |
| --- | --- | --- | --- |
| **RESULTS** | | |  |
| Study selection | 16a | Described in the results. |  |
|  | 16b | Described in the results. | Figure1 |
| Study characteristics | 17 | Described in the results. | Table1 |
| Risk of bias in studies | 18 | Described in the results. | Table 2 |
| Results of individual studies | 19 | Described in the results. | Figure2-3 |
| Results of syntheses | 20a | Described in the results. | Table 4-6 |
|  | 20b | Described in the results. |  |
|  | 20c | Described in the results. |  |
|  | 20d | Described in the results. |  |
| Reporting biases | 21 | Described in the results. |  |
| Certainty of evidence | 22 | Described in the results. |  |
| **DISCUSSION** | | |  |
| Discussion | 23a | Described in the discussion. |  |
|  | 23b | Described in the discussion. |  |
|  | 23c | Described in the discussion. |  |
|  | 23d | Described in the discussion. |  |
| **OTHER** **INFORMATION** | | |  |
| Registration and  protocol | 24a | The protocol is described in the method, we have registered in the Research Registry with PROSPERO (CRD42023414616). |  |
|  | 24b | PROSPERO |  |
|  | 24c | The protocol is described in the method. |  |
| Support | 25 | This work was supported by Health Commission of Hunan Province of China (No. 202114021174) and Changsha Science and Technology Bureau Hunan Province of China(No. kq1907068). |  |
| Competing interests | 26 | The authors declare that they have no competing interests. |  |
| Availability of data, code and other materials | 27 | The Supplementary Material. |  |

*From:* Page MJ, McKenzie JE, Bossuyt PM, Boutron I, Hoffmann TC, Mulrow CD, et al. The PRISMA 2020 statement: an updated guideline for reporting systematic reviews . BMJ 2021;372:n71. doi:

10. 1136/bmj.n71

For more information, visit: <http://www.prisma-statement.org/>
