# Supplementary material for: Dose-response relationships of resistance training in Type 2 diabetes mellitus: a meta-analysis of randomized controlled trials
Source: Front Endocrinol (Lausanne). 2023 Sep 25;14:1224161. doi: 10.3389/fendo.2023.1224161 (PMC10561623; doi:10.3389/fendo.2023.1224161)
Supplement: Supplementary file 2 [file Table_2.pdf]

Table S2 Search strategy for Embase

#	Searches
1	'resistance training'/exp
2	'training, resistance':ab,ti
3	'strength training':ab,ti
4	'weight-lifting exercise program':ab,ti
5	'weight lifting exercise program':ab,ti
6	'weight-lifting exercise programs':ab,ti
7	'weight-bearing strengthening program':ab,ti
8	'strengthening program, weight-bearing':ab,ti
9	'strengthening programs, weight-bearing':ab,ti
10	'weight bearing strengthening program':ab,ti
11	'weight-bearing strengthening programs':ab,ti
12	'weight-bearing exercise program':ab,ti
13	'exercise program, weight-bearing':ab,ti
14	'exercise programs, weight-bearing':ab,ti
15	'weight-bearing exercise programs':ab,ti
16	#1 OR #2 OR #3 OR #4 OR #5 OR #6 OR #7 OR #8 OR #9 OR #10 OR #11 OR #12 OR #13 OR #14 OR #15
17	'diabetes mellitus'/exp
18	'randomized controlled trial':ab,ti
19	'randomized':ab,ti
20	'double-blind':ab,ti
21	'placebo':ab,ti
22	#18 OR #19 OR #20 OR #21
23	#16 AND #17 AND #22
